# Choline Deficiency Drives the Inflammation–Fibrosis Cascade: A Spatiotemporal Atlas of Hepatic Injury from Weeks 6 to 10

**DOI:** 10.3390/antiox15010110

**Published:** 2026-01-15

**Authors:** Shang Li, Guoqiang Zhang, Xiaohong Li, Xu Zhao, Axi Shi, Qingmin Dong, Changpeng Chai, Xiaojing Song, Yuhui Wei, Xun Li

**Affiliations:** 1 The First School of Clinical Medicine, Lanzhou University, Lanzhou 730000, China; 120220903161@lzu.edu.cn (S.L.); zhaoxu2024@lzu.edu.cn (X.Z.); ldyy_danex@lzu.edu.cn (A.S.); dongqm2023@lzu.edu.cn (Q.D.); yhwei@lzu.edu.cn (Y.W.); 2 Department of General Surgery, The First Hospital of Lanzhou University, Lanzhou 730000, China; zhanggq15@lzu.edu.cn (G.Z.); chaichp08@163.com (C.C.); ldyy_songxjer@lzu.edu.cn (X.S.); 3 School of Pharmacy, Lanzhou University, Lanzhou 730000, China; lixh2024@lzu.edu.cn; 4 Medical Engineering Center for Liver Repair and Regeneration of Gansu Province, Lanzhou 730000, China; 5 Hepatopancreatobiliary Surgery Institute of Gansu Province, Lanzhou 730000, China; 6 Clinical Research Center for General Surgery of Gansu Province, Lanzhou 730000, China; 7 Innovation Center for Advanced Particle and Nuclear Medicine Technologies, The First Hospital of Lanzhou University, Lanzhou 730000, China; 8 Key Laboratory of Biotherapy and Regenerative Medicine of Gansu Province, Lanzhou 730000, China

**Keywords:** metabolic dysfunction-associated steatotic liver disease, choline deficiency, lipid metabolism, oxidative stress, mitochondria, inflammation

## Abstract

Metabolic dysfunction-associated steatotic liver disease (MASLD) is strongly linked to systemic metabolic disturbances and features a lipid-driven cascade that promotes hepatic inflammation and fibrosis. Choline insufficiency contributes to disease advancement by altering phospholipid turnover and redox homeostasis; however, its spatial and temporal regulatory roles throughout MASLD progression remain insufficiently defined. A 10-week high-fat, choline-deficient (HFCD) mouse model was established, and liver pathology was evaluated at weeks 6, 8, and 10. Time-resolved assessments combined untargeted metabolomics, magnetic resonance imaging–proton density fat fraction (MRI-PDFF), serum biochemistry, histological staining, immunofluorescence, and transmission electron microscopy to characterize dynamic alterations in lipid metabolism, redox status, inflammation, and fibrogenesis. The HFCD diet produced a clear temporal sequence of liver injury. Steatosis, phosphatidylcholine depletion, and early antioxidant loss appeared by week 6. By week 8, mitochondrial structural damage and pronounced cytokine elevation were evident. At week 10, collagen deposition and α-SMA activation signaled fibrotic progression. Metabolomics indicated significant disruptions in pathways related to ATP-binding cassette (ABC) transporters, one-carbon metabolism, and the tricarboxylic acid (TCA) cycle. Using integrated analytical strategies, this study suggests that choline deficiency may be associated with a time-dependent pathological cascade in MASLD, beginning with phospholipid destabilization and extending to altered mitochondria–endoplasmic reticulum crosstalk at mitochondria-associated membranes, alongside amplified oxidative–inflammatory responses, which collectively may contribute to progressive fibrogenesis as the disease advances.

## 1. Introduction

Metabolic dysfunction-associated steatotic liver disease (MASLD), the updated nomenclature replacing non-alcoholic fatty liver disease (NAFLD), encompasses a broad pathological spectrum initiated by disturbances in hepatic lipid metabolism, evolving from simple fat deposition to hepatic inflammation, fibrosis, and end-stage cirrhosis [1,2]. In 2023, three international hepatology societies proposed replacing the term NAFLD with MASLD [3]. This updated nomenclature highlights the central role of metabolic dysfunction in disease pathogenesis [4]. The diagnosis of MASLD is confirmed when hepatic steatosis is present in conjunction with at least one risk factor—such as obesity, type 2 diabetes mellitus, or hypertension—after excluding other potential causes of hepatic fat accumulation [5,6].

MASLD is among the most widespread hepatic disorders globally and has already affected the health of a substantial proportion of the population—over one-third worldwide. Recent systematic reviews and meta-analyses indicate that the global prevalence of this condition has risen from 25.3% to 38.0%. With ongoing shifts in dietary habits driven by worldwide economic development, MASLD is projected to become the leading cause of chronic liver disease by 2030 [7,8]. MASLD contributes not only to hepatic complications—including cirrhosis, terminal liver failure, and hepatocellular carcinoma—but also increases susceptibility to a range of extrahepatic disorders, such as cardiovascular disease, chronic kidney disease, and certain malignancies beyond the liver. Epidemiological evidence shows that nearly 60% of individuals with MASLD eventually succumb to non-hepatic causes—predominantly cardiovascular disease and extrahepatic malignancies (with diabetes-related complications also contributing)—highlighting its multisystemic nature and its growing burden on global health [9,10].

Although the nomenclature of this disease spectrum has evolved over time, its pathological essence consistently originates from hepatic lipid metabolic imbalance [11]. Lipid overloading in hepatocytes induces a buildup of reactive oxygen species (ROS), compromising mitochondrial function and causing lipid peroxidation [12]. This sequence of events ultimately triggers an oxidative stress-mediated inflammatory cascade. This process ultimately drives hepatic stellate cell activation and extracellular matrix deposition, resulting in progressive fibrosis—the strongest predictor of liver-related mortality [13,14].

Choline metabolism is closely associated with hepatic lipid handling and redox balance. As choline is essential for phosphatidylcholine synthesis—supporting hepatocellular membrane integrity—for very-low-density lipoprotein (VLDL) assembly/secretion to enable lipid export, reduced choline availability is often viewed as a plausible contributor to lipid retention and oxidative imbalance [15,16,17]. In line with this view, prior studies have reported that choline insufficiency commonly accompanies hepatic lipid accumulation, increased ROS levels, and diminished activities of antioxidant enzymes such as superoxide dismutase (SOD) and glutathione peroxidase (GPx), changes that may promote inflammatory activation and fibrogenic remodeling [16,18,19,20]. Nevertheless, the current literature is largely associative, and the temporal sequence of these events during HFCD feeding remains incompletely characterized. Longitudinal datasets that map lipidomic perturbations, bioenergetic compromise, inflammation propagation, and stromal restructuring onto clearly staged pathological landmarks are still limited, highlighting the need for time-resolved studies integrating metabolic and multi-scale phenotyping. Therefore, elucidating the dynamic spatiotemporal patterns potentially linking choline deficiency to the inflammation–fibrosis continuum under a high-fat diet may help refine our understanding of MASLD progression. Employing a multi-omics integrative framework, we aimed to characterize stage-dependent metabolic remodeling in HFCD-fed mice, with a particular focus on choline metabolism, phospholipid homeostasis, organelle stress responses (including altered mitochondria–endoplasmic reticulum crosstalk at mitochondria-associated membranes, MAMs), and ROS-associated inflammatory and fibrogenic signaling.

## 2. Materials and Methods

### 2.1. Animal Procedures

Thirty-six male C57BL/6J mice, free of specific pathogens (SPF grade), aged 6–8 weeks and weighing 18–20 g, were procured from the Lanzhou Veterinary Research Institute of the Chinese Academy of Agricultural Sciences (Lanzhou, Gansu, China; production license SCXK [Gan] 2020-0002). Animals were accommodated in the Good Laboratory Practice-approved animal facility at Lanzhou University, where SPF standards were strictly observed. Environmental parameters were kept stable, with room temperature maintained at 22 ± 2 °C and relative humidity at 50–60%. The housing area followed a 12 h light–dark rhythm (illumination from 07:00 to 19:00), provided 10–20 air exchanges per hour, and ensured background noise levels did not exceed 60 dB.

Following a seven-day acclimatization, six mice were randomly selected for the control group with a standard diet, and the remaining thirty mice were placed on a HFCD diet to establish the model. All animals had free access to food and water. Based on results from preliminary experiments, starting from week six, animals were sacrificed in batches over five consecutive weeks, with six mice randomly selected each week until week ten. Prior to sample collection, mice were fasted for eight hours. Following anesthesia, blood samples were collected via retro-orbital puncture. Portions of the left lateral hepatic lobe were used for transmission electron microscopy, and left lateral and left median hepatic lobes were rapidly frozen in liquid nitrogen and kept at −80 °C for downstream analytical measurements. The right lateral lobe was fixed in 4% paraformaldehyde for histopathological examination, and the right median lobe was used for Oil Red O staining after frozen section. All experimental procedures involving animals were performed in accordance with the ethical guidelines and welfare regulations of the Institutional Animal Care and Use Committee of the First Hospital of Lanzhou University (approval number: LDYYLL2023-544), adhering to the 3R principles of replacement, reduction, and refinement.

### 2.2. Diet Composition

The HFCD diet was obtained from Keao Xieli (Tianjin) Feed Co., Ltd. (Tianjin, China). The detailed formulation is shown in Table 1. The HFCD formulation was designed with a macronutrient caloric ratio of 60% lipid-derived energy (primarily from lard), 20% carbohydrate energy (maltodextrin-based), and 20% protein energy (casein as primary source) (Table 1). The energy density of the HFCD was 5200 kcal/kg (i.e., 5.2 kcal/g), according to the manufacturer. The standard chow diet (maintenance diet for rats/mice; SPF Biotechnology, Beijing, China; Cat. No. SPF-F02-002) had an estimated energy density of ~3538 kcal/kg, with ~66.3% carbohydrate, ~22.0% protein, and ~11.7% fat-derived energy.

### 2.3. Biochemical Analyses

Hepatic biochemical parameters were determined in liver tissue homogenates. Biochemical profiling encompassed lipid metabolism markers (triglycerides (TG), total cholesterol (TC), VLDL, and high-density lipoprotein (HDL)) and hepatocellular injury indices (aspartate aminotransferase (AST) and alanine aminotransferase (ALT)). Concurrently, oxidative stress parameters were evaluated through enzymatic activity assays for SOD, GPx, catalase (CAT), and the lipid peroxidation product malondialdehyde (MDA). All colorimetric analyses were performed using commercially available kits (Nanjing Jiancheng Bioengineering Institute, Nanjing, China) strictly adhering to the manufacturer’s standardized protocols.

### 2.4. Measurement of Inflammatory Cytokines

Hepatic levels of inflammatory cytokines, including interleukin-6 (IL-6) and tumor necrosis factor-alpha (TNF-α), were determined using ELISA kits obtained from Wuhan Elabscience Biotechnology Co., Ltd. (Wuhan, China). All assays were conducted following the manufacturer’s instructions.

### 2.5. Histopathological Analysis of Liver Tissue

Liver tissues from the right lobe were fixed in 4% paraformaldehyde for 24 h at 4 °C, paraffin-embedded, and sectioned at 5 μm. H&E staining was performed to assess general histology, while Masson’s trichrome and Sirius Red (0.1% in saturated picric acid) staining were used to visualize fibrotic alterations. For assessment of lipid accumulation, cryosections (18 μm) from frozen liver specimens were prepared on a Thermo Fisher cryostat (Waltham, MA, USA) and stained with Oil Red O, followed by hematoxylin counterstaining. Stained slides were observed under a Nikon Ti2 light microscope (Nikon, Tokyo, Japan).

### 2.6. Ultrastructural Observation via Transmission Electron Microscopy (TEM)

Approximately 1 mm^3^ fragments of fresh hepatic tissue were rapidly excised (<3 min post-dissection) and fixed in an electron microscopy fixative solution. After fixation, the specimens underwent conventional processing involving dehydration, resin infiltration, embedding, and polymerization, followed by trimming. Ultrathin sections of 60–80 nm were produced using a diamond knife and subsequently stained with uranyl acetate (2% in 50% ethanol) and lead citrate. The ultrastructural morphology of hepatocytes—including mitochondria, endoplasmic reticulum, and lipid droplets—was visualized with a Hitachi HT7700 TEM (Hitachi, Tokyo, Japan).

### 2.7. Immunofluorescence Analysis

Liver specimens were fixed in 4% PFA (pH 7.4; 4 °C, 24 h), embedded in paraffin, and sectioned into 4 μm slices. Deparaffinized and rehydrated sections underwent antigen retrieval in sodium citrate buffer (0.01 M, pH 6.0) using 95 °C heat mediation for 20 min. After PBS rinsing, nonspecific binding was blocked with 5% serum/1% BSA solution prior to sequential incubations: primary antibodies (4 °C, 16 h) followed by fluorophore-conjugated secondary antibodies (RT, 1 h; dark conditions). Nuclei were counterstained with 4′,6-diamidino-2-phenylindole (DAPI) and mounted using antifade medium. Fluorescence signals were visualized with a confocal laser scanning microscope under identical exposure settings for all samples. Both positive and negative controls were included to validate staining specificity.

### 2.8. MRI–PDFF Analysis

To ensure data consistency and minimize gastrointestinal interference, all mice underwent an 8 h fasting period before the magnetic resonance imaging (MRI) procedures. Anesthesia was initiated using 5% isoflurane delivered in a gaseous mixture of 70% N_2_O and 30% O_2_, and was subsequently sustained with 1.5–2% isoflurane through a face mask. The MRI–proton density fat fraction (PDFF) acquisitions were performed on a uMR 9.4T preclinical system (manufactured by United Imaging Life Sciences Instruments Co., Ltd., Wuhan, China), which was fitted with a specialized radiofrequency coil designed for small-animal abdominal imaging. A multi-echo gradient echo (ME-GRE) sequence was employed for PDFF quantification, configured with the following parameters: six echoes with echo times (TEs) incrementing from 1.0 to 12.0 ms; a repetition time (TR) of approximately 120 ms; a flip angle of 12°; a field of view (FOV) of 40 × 40 mm; a matrix size of 128 × 128; a slice thickness of 1.0 mm; and an interslice gap of 0.1 mm.

Acquired images were analyzed using vendor-provided software to calculate the hepatic PDFF via a fat–water separation algorithm. For quantitative analysis, 3–5 slices were randomly selected per animal, with five evenly distributed regions of interest (ROIs, ~1 mm^2^ each) per slice, avoiding large vessels and tissue edges. The mean PDFF value from all ROIs was used as the final measurement for each mouse. All measurements were independently performed by two experienced investigators under blinded conditions.

### 2.9. Untargeted Metabolomics

Frozen liver tissues stored at −80 °C were thawed on ice and homogenized in extraction solvent (MeOH:ACN:H_2_O = 2:2:1, *v*/*v*/*v*) containing deuterated internal standards. After centrifugation (13,800× *g*, 15 min, 4 °C), the supernatants were collected for LC–MS/MS analysis. A pooled QC sample was prepared by mixing equal aliquots from all samples and injected periodically to monitor instrument stability. Polar metabolites were analyzed using a Vanquish UHPLC system coupled to an Orbitrap Exploris 120 mass spectrometer (Thermo Fisher Scientific) equipped with a Waters ACQUITY UPLC BEH Amide column (2.1 × 50 mm, 1.7 μm). The mobile phase consisted of 25 mmol/L ammonium acetate and 25 mmol/L ammonia hydroxide in water (pH 9.75) (A) and acetonitrile (B). Data acquisition was controlled by Xcalibur (v4.4, Thermo Fisher Scientific). Raw files were converted to mzXML using ProteoWizard (v3.0.24054) and processed using an in-house R pipeline based on XCMS (R v4.3.2, XCMS v3.18.0) for feature detection, alignment, and normalization. Metabolite identification was performed by accurate mass and MS/MS spectral matching, with database referencing to HMDB, METLIN, and KEGG. Detailed metabolite annotation and database-assisted identification procedures are provided in Supplementary Methods S1 and S2.

### 2.10. Statistical Analysis

All data are presented as mean ± SD. Statistical analyses were conducted using GraphPad Prism 10.0 (GraphPad Software, La Jolla, CA, USA), ImageJ, 1.54g (National Institutes of Health, Bethesda, MD, USA) and Image-Pro Plus 6.0.0.260 (Media Cybernetics, Inc., Rockville, MD, USA). For comparisons between two groups, paired Student’s *t*-tests were applied. When multiple groups were compared, one-way ANOVA with Tukey’s post hoc test was used, and Holm–Sidak correction was applied as needed. A significance threshold of *p* < 0.05 was adopted.

## 3. Results

### 3.1. General Conditions After HFCD Feeding

**Figure 1:**
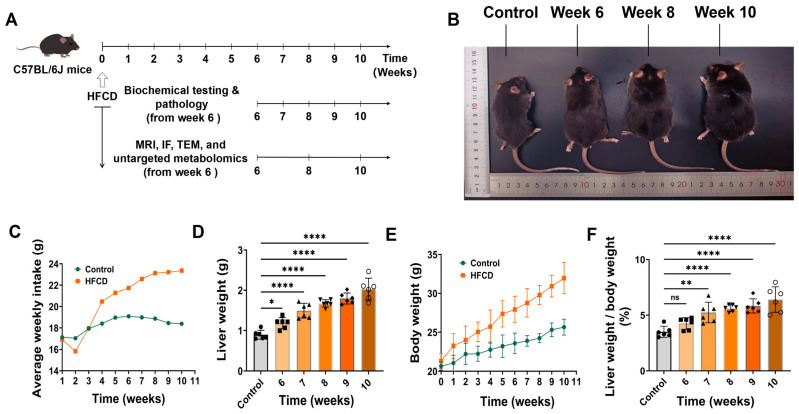
Changes in body weight, food intake, liver weight, and liver-to-body weight ratio in C57BL/6J mice under different dietary conditions. (**A**) Both HFCD and control groups were subjected to 10 weeks of intervention. Biochemical and histopathological analyses were performed weekly starting from week 6, while MRI–PDFF and untargeted metabolomics were conducted every two weeks. (**B**) Representative macroscopic differences in body morphology between mice in the HFCD and control groups. (**C**) Average weekly food intake at different modeling stages. (**D**) Changes in body weight throughout the modeling period. (**E**) Liver weight and (**F**) liver-to-body weight ratio at different time points starting from week 6. Data are presented as mean ± SD (*n* = 6, ns, not significant,* *p* < 0.05, ** *p* < 0.01, **** *p* < 0.0001).

During the 10-week period of HFCD diet administration, the HFCD-fed mice exhibited noticeable differences in body size compared with the control group. Macroscopically, variations in body morphology became increasingly evident across different time points. The average food intake per mouse showed no significant difference between the two groups during the first and third weeks; however, a marked decline was observed in the second week, followed by a gradual increase thereafter, displaying a distinct temporal pattern (Figure 1B,C). Weekly body weight measurements revealed a significantly higher weight gain trend in the HFCD group compared with controls (Figure 1D). Statistical analysis of liver weight and the liver-to-body weight ratio at different time points demonstrated that both parameters began to diverge significantly from week 6. 

### 3.2. Time-Dependent Development of Hepatic Steatosis and Fibrosis in HFCD-Fed Mice

**Figure 2:**
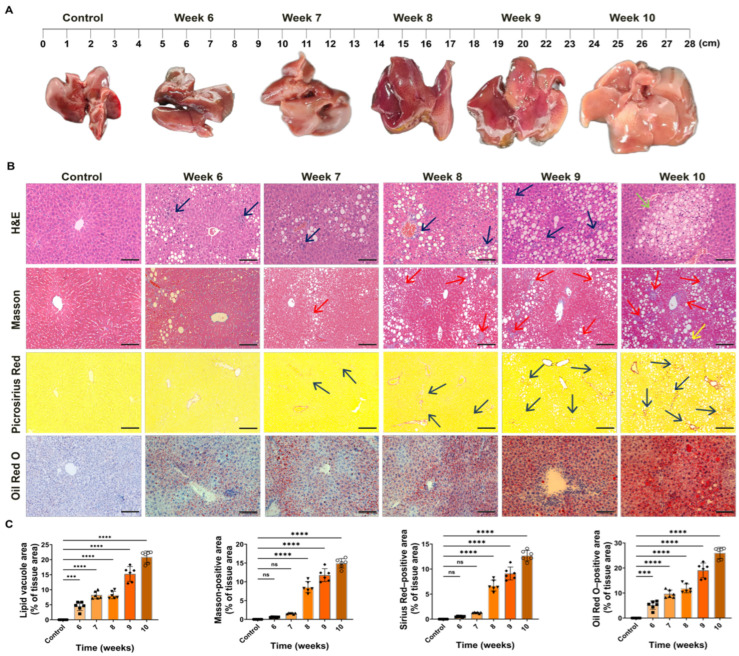
Representative liver morphology and histopathological features of mice in the control and HFCD-fed groups at different time points. (**A**) Macroscopic appearance of livers from the control group and HFCD-fed mice at weeks 6, 7, 8, 9, and 10 following a 10-week feeding regimen. (**B**) Histological staining of liver tissues at each time point (H&E, Masson’s trichrome, Picrosirius Red, and Oil Red O; magnification 200×, scale bar = 100 μm). Arrows indicate key histopathological features: blue arrows, ballooning degeneration with macrophage infiltration; green arrows, focal necrotic foci; red arrows, bridging fibrosis on Masson’s trichrome staining (central–central or central–portal pattern); black arrows, collagen deposition/fibrotic areas on Picrosirius Red staining; yellow arrows, representative fibrotic/lesion regions highlighted at later time points. (**C**) Quantification of lipid vacuole area (H&E), Masson-positive area, Sirius Red-positive area, and Oil Red O-positive area (% tissue area) at weeks 6–10 (*n* = 6). (ns, not significant; *** *p* < 0.001, **** *p* < 0.0001).

During the 10-week HFCD feeding period, macroscopic examination revealed progressive morphological alterations in the liver. By week 6, the livers of HFCD-fed mice appeared enlarged and dark red in color but retained a glossy surface. As feeding continued, the liver volume further increased, the surface luster diminished, and the edges became rounded. By week 10, the livers exhibited a yellowish-brown coloration, softer texture, and a greasy appearance to the touch (Figure 2A).

To further characterize hepatic pathology, four histological staining methods were applied. H&E staining revealed ballooning degeneration accompanied by macrophage infiltration as early as week 6 (indicated by blue arrows). The extent of ballooning and macrophage aggregation increased progressively, and by week 10, focal necrotic lesions were evident (green arrows). Masson’s trichrome staining showed thin, filamentous blue collagen fibers around the portal area at week 6, without forming continuous fibrous septa. From week 7 onward, collagen fibers extended from the portal tract into the hepatic lobules, forming a “stellate” fibrotic network that began to partition the parenchyma into early pseudolobular structures. By week 10, bridging fibrosis connecting central veins and portal tracts (“central–central” or “central–portal” patterns) was observed (red arrows). Picrosirius Red staining further confirmed progressive collagen deposition, showing an increase in reddish-orange fibrotic areas from weeks 8–10, consistent with the Masson’s results. Oil Red O staining revealed small, evenly distributed red lipid droplets around central veins at week 6. From weeks 8 to 10, these lipid droplets coalesced into large aggregates occupying more than 90% of the cytoplasm, forming marked macrovesicular steatosis with occasional crystallized free fatty acids in the cytoplasm.

Quantitative image analysis revealed a time-dependent increase in hepatic lipid accumulation and fibrotic deposition during HFCD feeding (Figure 2C). Compared with controls, the lipid vacuole area on H&E staining and the Oil Red O-positive area progressively rose from weeks 6 to 10, with more pronounced differences emerging at the later time points. In parallel, collagen deposition quantified by Masson’s trichrome and Picrosirius Red staining increased across the time course, showing minimal changes at the early stages but marked elevation toward weeks 9–10. Collectively, these staining techniques demonstrated that HFCD feeding induced hepatic lipid accumulation beginning at week 6, accompanied by fibrosis development and inflammatory cell infiltration—a process involving steatosis, inflammation, and hepatocellular injury. By week 10, histopathological features were consistent with steatohepatitis.

### 3.3. HFCD Feeding Induces Hepatic Lipid Metabolic Disorders Accompanied by Inflammation

**Figure 3:**
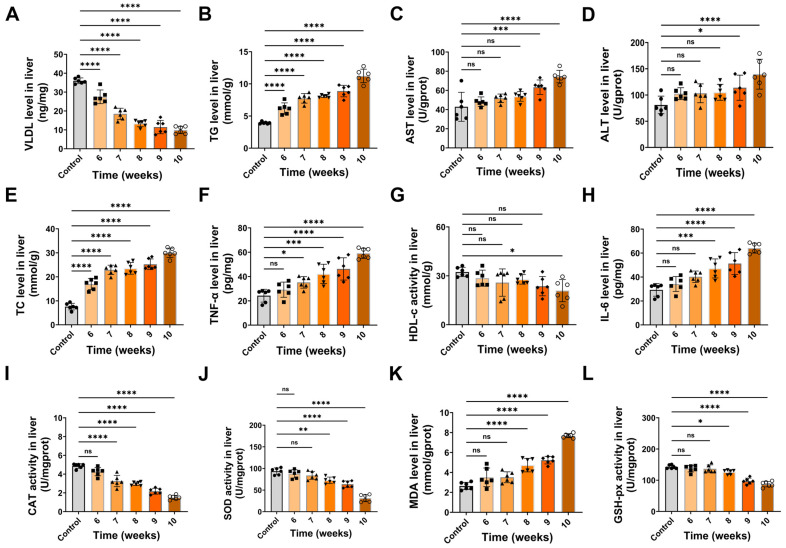
Biochemical profiles of hepatic tissues from C57BL/6J mice under different dietary conditions at various time points. (**A**) VLDL level; (**B**) TG level; (**C**) AST level; (**D**) ALT level; (**E**) TC level; (**F**) TNF-α level; (**G**) HDL-c activity; (**H**) IL-6 level; (**I**) CAT activity; (**J**) SOD activity; (**K**) MDA level; (**L**) GSH-Px activity. Data are presented as mean ± SD (*n* = 6). ns, not significant; * *p* < 0.05, ** *p* < 0.01, *** *p* < 0.001, **** *p* < 0.0001.

According to histopathological evaluation, biochemical assays were performed on hepatic tissues collected from HFCD-fed mice at various time points and compared with those from the control group. Based on liver homogenate measurements, VLDL showed a progressive decrease over time, with values lower than controls from week 6 onward and the lowest levels at weeks 9–10 (Figure 3A). TG increased in a time-dependent manner and peaked at week 10 (Figure 3B). AST remained largely unchanged at weeks 6–8 but rose at weeks 9–10 (Figure 3C), and ALT showed mostly non-significant changes early but a higher level at the late stage, particularly at week 10 (Figure 3D). TC was elevated from week 6 and continued to increase through week 10 (Figure 3E). TNF-α increased gradually, with more apparent elevations at weeks 9–10 (Figure 3F). HDL-C displayed an overall downward trend, with differences becoming more evident at later time points (Figure 3G). IL-6 increased over time and was higher at weeks 9–10 (Figure 3H). CAT and SOD activities declined progressively, with more pronounced reductions toward week 10 (Figure 3I,J). In parallel, MDA increased over the course of feeding, with the largest rise at week 10 (Figure 3K), whereas GSH-px showed a gradual decline with lower levels at later time points (Figure 3L). Collectively, these results indicate that HFCD feeding induces lipid metabolic dysregulation, oxidative stress, and an inflammatory cascade, leading to lipotoxic hepatic injury. Moreover, this pathological progression intensifies progressively with prolonged dietary intervention.

### 3.4. MRI and Immunofluorescence Analyses Further Confirmed That HFCD Feeding Induces Hepatic Lipid Accumulation, Inflammation, and Fibrosis

**Figure 4:**
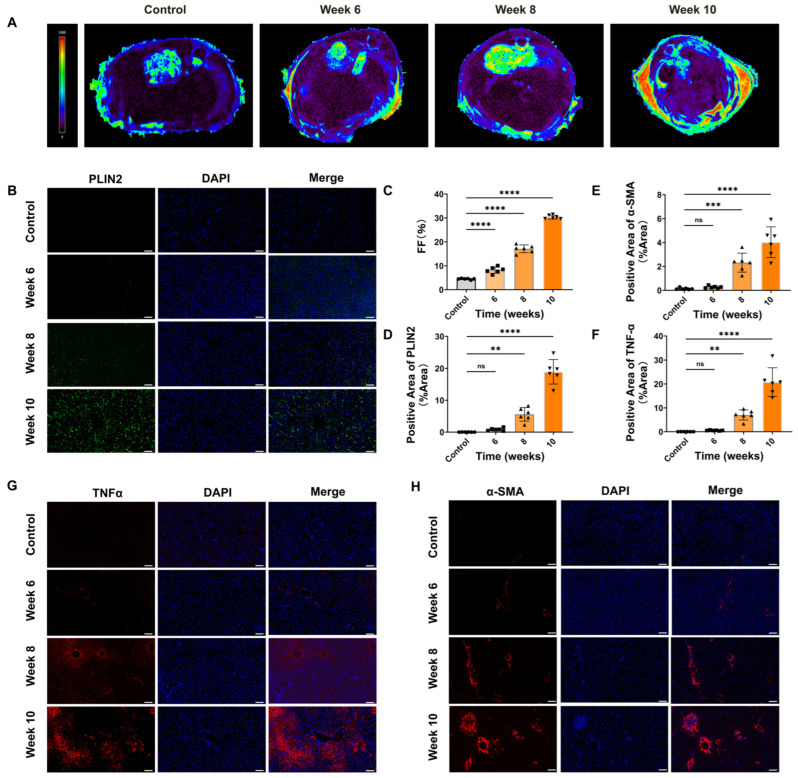
Comparative analysis of hepatic lipid accumulation, inflammation, and fibrosis between the two dietary groups using MRI–PDFF and immunofluorescence techniques. (**A**,**C**) MRI–PDFF images and corresponding quantitative analysis showing hepatic fat content in control and HFCD-fed mice (n = 3). (**B**) Immunofluorescence visualization of PLIN2 expression across different time points compared with the control group. (**D**–**F**) Quantitative statistical analysis of the fluorescent signal areas of PLIN2, TNF-α, and α-SMA across different feeding periods. (**G**) TNF-α fluorescence expression in liver tissues over time compared with the control group. (**H**) α-SMA fluorescence expression across different feeding durations. Scale bar = 100 μm. Data are expressed as mean ± SD (*n* = 6). ns, not significant; ** *p* < 0.01, *** *p* < 0.001, **** *p* < 0.0001.

To further validate the occurrence of lipid accumulation, inflammation, and fibrosis during the HFCD feeding period, MRI–PDFF analysis was performed. Compared with the control group, hepatic fat accumulation was markedly increased in HFCD-fed mice, reaching approximately 30% by week 10 (*p* < 0.05) (Figure 4A,C). Consistently, PLIN2 immunofluorescence revealed progressively enhanced fluorescence intensity, indicating elevated lipid droplet content, with significant differences among groups (Figure 4B,D). Furthermore, immunofluorescence assessment of α-SMA and TNF-α expression demonstrated that fluorescence intensity in the control group was markedly lower than that in the HFCD group (Figure 4E–H), suggesting activation of hepatic stellate cells and inflammatory signaling during HFCD-induced liver injury.

### 3.5. Mitochondrial Alterations Under HFCD Feeding Revealed by Transmission Electron Microscopy and Immunofluorescence

**Figure 5:**
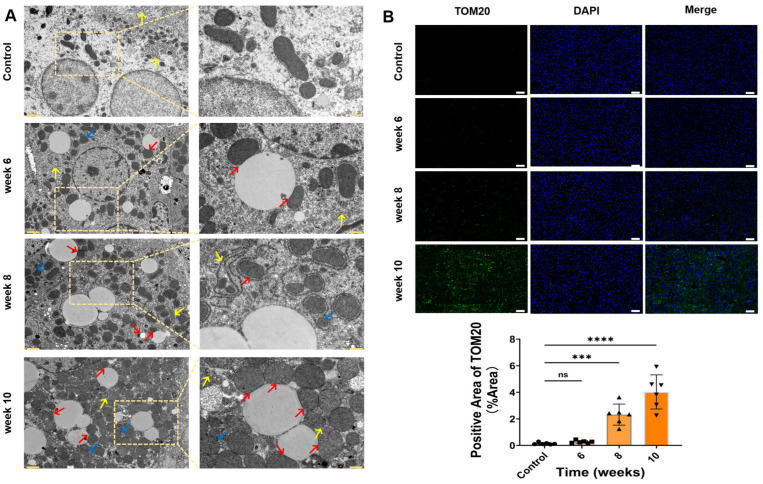
Transmission electron microscopy (TEM) and immunofluorescence analyses comparing hepatic ultrastructure and mitochondrial morphology in mice under different dietary regimens across various time points. (**A**) Representative TEM images of liver tissues. Red arrows indicate swollen and morphologically abnormal mitochondria in contact with lipid droplets; yellow arrows denote dilated endoplasmic reticulum (ER) with lipid droplet infiltration; blue arrows highlight clusters of morphologically abnormal mitochondria (scale bar: left = 1.25 µm, right = 5 µm). (**B**) Immunofluorescence analysis of TOM20 expression in liver tissues (scale bar = 100 µm). (Data are expressed as mean ± SD (*n* = 6). ns, not significant; *** *p* < 0.001, **** *p* < 0.0001).

Compared with the control group, livers from HFCD-fed mice exhibited progressive mitochondrial abnormalities over time. Mitochondria appeared increased in both number and size, with disrupted or indistinct cristae, and were frequently observed in close contact with enlarged lipid droplets. Additionally, hepatocytes displayed proliferation and thickening of the endoplasmic reticulum (ER), with more extensive branching and dilation (Figure 5A). Immunofluorescence analysis of TOM20, a mitochondrial outer membrane marker, revealed progressively enhanced fluorescence intensity during HFCD feeding, suggesting changes in mitochondrial abundance and/or organization (Figure 5B).

### 3.6. Global Metabolomic Profiling and Comparative Analysis of Differential Metabolites

**Figure 6:**
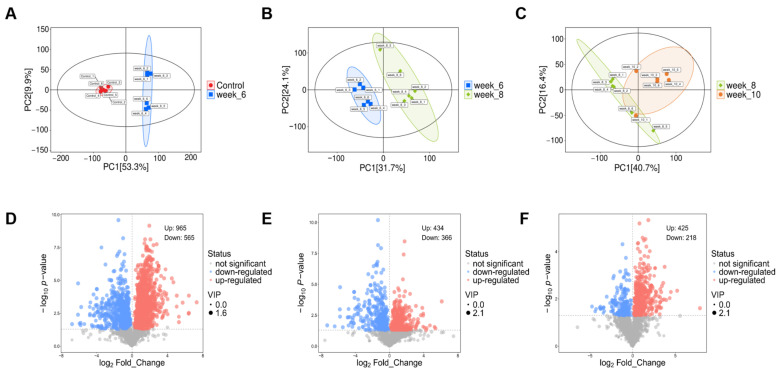
(**A**–**C**) PCA score plots showing metabolic profile separation across groups. PC1 and PC2 (axes labeled with variance contribution rates) illustrate intergroup discrimination. Samples are color-coded by group, and each symbol represents one biological replicate. Pooled QC samples were injected periodically to monitor instrument stability and were included in the PCA visualization as QC points. (**D**–**F**) Volcano plots showing differential metabolite distributions in mouse liver tissues between comparison groups. Each dot represents one metabolite; red and blue indicate significantly increased and decreased metabolites, respectively, and gray indicates non-significant metabolites. The x-axis shows the log2 fold change (log2FC), and the y-axis shows the −log10(*p*-value). The vertical dashed line indicates log2FC = 0, and the horizontal dashed line indicates the significance threshold (*p* = 0.05; −log10 *p* = 1.30). Differential metabolites were screened using VIP > 1 and *p* < 0.05; dot size corresponds to the VIP score (n = 6 biological replicates per group). (*n* = 6).

Visual inspection of the data revealed that the unsupervised principal component analysis (PCA) model clearly distinguished different metabolic phenotypes based on their unique metabolite distribution patterns. Cross-group comparisons using the supervised partial least squares discriminant analysis (PLS-DA) model demonstrated an effective separation of phenotypes, with high model fitness (R^2^) and excellent predictive performance (Q^2^). Based on the results of three pairwise comparisons and predefined screening criteria, multiple differential metabolites were successfully identified, as illustrated in Figure 6A–C. Comparative metabolomic analysis across different time points revealed extensive metabolic remodeling. Specifically, between the control and HFCD groups at week 6, 965 metabolites were upregulated and 565 were downregulated. Between weeks 8 and 6, 434 were upregulated and 366 downregulated, while comparison between weeks 10 and 8 identified 425 upregulated and 218 downregulated metabolites (Figure 6D–F).

### 3.7. Potential Metabolic Pathways Associated with Disease Progression

**Figure 7:**
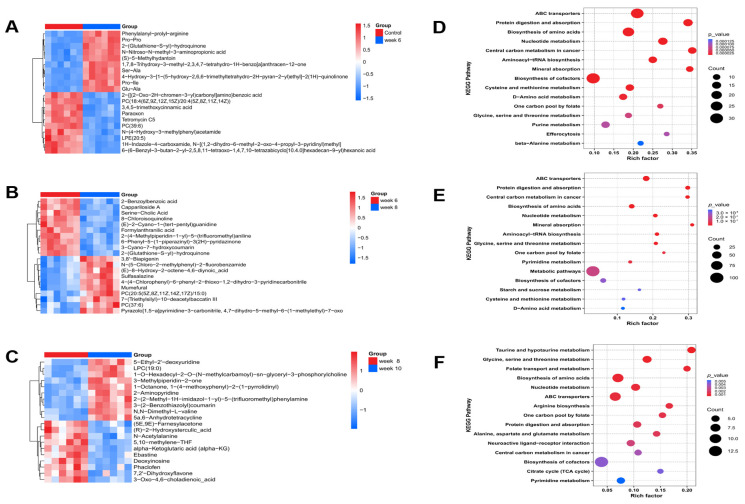
(**A**–**C**) Heatmaps illustrating the relative abundance profiles of significantly altered metabolites across different experimental groups. The x-axis denotes the respective treatment groups, and the y-axis lists metabolites that were significantly changed between comparisons. Each cell reflects the normalized abundance level of a metabolite, where red indicates higher and blue indicates lower relative concentrations within each group. (**D**–**F**) KEGG pathway enrichment analysis presented as bubble plots. Enrichment analysis revealed key pathways (y-axis) with x-axis representing Rich Factor (differential/total metabolites per pathway), bubble size proportional to metabolite count, and color gradient, warm reflects increasing statistical significance reflecting statistical significance. (*n* = 6).

To illustrate the relative abundance patterns of differential metabolites, the normalized metabolomic dataset was subjected to unit variance (UV) scaling followed by hierarchical clustering analysis using the ComplexHeatmap package (v2.22.0) in R (v4.3.2). For each comparison, Euclidean distance matrices were computed, and clustering was carried out via the complete linkage algorithm. The ten most significantly upregulated and downregulated metabolites were visualized in heatmaps, suggesting distinct alterations potentially involving phospholipid remodeling, mitochondrial energy production, nucleotide turnover, oxidative stress responses, bile acid metabolism, peptide processing, and fibrosis-related signaling pathways (Figure 7A–C). Furthermore, KEGG pathway enrichment analysis was performed to identify pathways that may be associated with these differential metabolites. The enrichment level, represented by the Rich Factor (i.e., the proportion of differential metabolites annotated to a specific pathway relative to all metabolites within that pathway), was used to assess pathway enrichment—higher Rich Factor values indicated stronger statistical enrichment. As an annotation- and statistics-based approach, pathway enrichment does not by itself demonstrate pathway activation or causality. Metabolic pathway enrichment analysis indicated enrichment in ABC transporters, protein digestion/absorption, amino acid biosynthesis (cysteine/methionine, glycine/serine/threonine, alanine/aspartate/glutamate), nucleotide metabolism, central carbon metabolism-related pathways, neuroactive ligand–receptor interaction, autophagy-related regulation, and TCA cycle–related processes (Figure 7D–F). Collectively, these results provide an exploratory overview of pathway-level associations that may help prioritize targets for subsequent validation.

## 4. Discussion

Overall, this study, through a 10-week temporal observation, demonstrated that a HFCD diet efficiently induces typical pathological alterations characteristic of MASLD in mice. Commencing at the sixth experimental week, a marked rise in both liver mass proportion (relative to body weight) and proton density fat fraction was observed, along with Oil Red O staining showing extensive lipid droplet accumulation, indicated the onset of hepatic steatosis. As the disease progressed to weeks 8–10, H&E staining revealed inflammatory cell infiltration and necrotic foci, while Masson’s trichrome and Sirius Red staining showed the expansion of collagen fiber networks, consistent with the pathological transition from steatosis to fibrosis.

This time-resolved model not only recapitulates the histological spectrum of MASLD but also provides a useful framework to dissect its dynamic molecular mechanisms. Our findings indicate that HFCD feeding is associated with marked steatosis within six weeks, accompanied by evidence of oxidative stress and inflammatory activation by weeks 7–8, ultimately progressing to a steatohepatitis-like phenotype with bridging fibrosis by week 10.

Notably, oxidative stress appeared to precede overt inflammation. The activities of SOD, GSH-Px, and CAT began to decline as early as week 6, accompanied by a simultaneous rise in MDA levels, suggesting that mitochondrial dysfunction and impaired free radical scavenging may represent early pathological events. These findings were corroborated by electron microscopy, which revealed mitochondrial swelling, cristae disruption, and close contacts between lipid droplets and mitochondria—indicating that lipotoxicity may impair organelles involved in energy metabolism. Meanwhile, the continuous upregulation of PLIN2 was consistent with increased lipid droplet stability and lipid storage burden, whereas the elevation of TNF-α and IL-6 levels at weeks 7–8 was consistent with activation of inflammatory responses, potentially involving Kupffer cell recruitment and amplification of the inflammatory cascade.

A central contribution of this study lies in highlighting the potential role of choline metabolic dysregulation in the pathogenesis of MASLD within the HFCD model. Choline serves as an essential substrate for the synthesis of phosphatidylcholine (PC) and very low-density lipoprotein (VLDL). Reduced choline availability not only may compromise hepatocellular membrane integrity but also may impair triglyceride export, thereby favoring intracellular lipid accumulation [21,22]. Moreover, previous studies have shown that choline participates in the methyl donor cycle (via betaine), thereby influencing DNA methylation and gene expression regulation [23]. In our metabolomic analysis, enrichment of the glycine/serine/threonine metabolism pathway was observed, which may be compatible with this mechanism, although direct evidence is not available in the current study. More importantly, choline deficiency has been linked to weakened hepatic antioxidant defense in prior reports. Literature reports have indicated that choline depletion may suppress antioxidant enzyme expression via Nrf2-related signaling [21,24,25]. Consistent with this, we observed decreased activities of SOD and GSH-Px in the HFCD group, which is consistent with this mechanistic hypothesis. Therefore, we suggest that choline-related perturbations may act as a contributor to lipid accumulation and may facilitate amplification of the oxidative stress–inflammation axis.

Untargeted metabolomic profiling further broadened the mechanistic perspective of this study. KEGG enrichment analysis suggested that differential metabolites were predominantly associated with pathways involved in phospholipid metabolism, the tricarboxylic acid (TCA) cycle, amino acid metabolism, and oxidative stress-related processes. Alterations in TCA cycle intermediates were consistent with potential mitochondrial energy metabolism perturbation, aligning with our electron microscopy observations. Additionally, enrichment of pathways such as ABC transporters and protein digestion and absorption was observed, which may reflect broader changes in hepatic nutrient handling and metabolic homeostasis. Collectively, these findings suggest that HFCD feeding is accompanied by multi-pathway metabolic remodeling affecting energy supply, membrane lipid homeostasis, and redox balance, with the enrichment results interpreted as pathway-level associations pending targeted confirmation.

Hepatic lipid homeostasis relies, at least in part, on adequate phosphatidylcholine (PC) availability, which contributes to VLDL assembly and lipid export. As a substrate for PC synthesis through the CDP-choline (Kennedy) pathway, reduced choline availability may limit hepatic PC production, thereby constraining VLDL assembly/secretion and favoring intracellular lipid retention. Interestingly, the concurrent increase in several dipeptide and tripeptide metabolites (e.g., phenylalanyl–prolyl–arginine) may indicate early perturbations in protein turnover, potentially reflecting a compensatory adaptation under lipotoxic conditions. Consistent with this interpretation, electron microscopy at week 6 showed mild endoplasmic reticulum (ER) dilation, which is compatible with an early ER stress response. In addition, enrichment signals related to folate-dependent one-carbon metabolism suggest that HFCD feeding may be accompanied by shifts in methyl-group metabolism and broader amino acid/nucleotide networks, although direct choline/PC quantification and targeted validation were not performed. Collectively, these findings align with an early stage characterized by metabolic perturbation and compensatory remodeling, preceding more overt inflammatory and fibrogenic features later in the time course.

In terms of lipid metabolism, lipid overload not only promotes steatosis but also triggers oxidative stress and organelle injury through lipotoxicity [26]. In this study, at week 8, metabolomic analysis showed downregulation of TCA cycle intermediates such as α-ketoglutarate, along with altered oxidative stress-related glutathione conjugates (e.g., 2-(Glutathione-S-yl)-hydroquinone), indicating disrupted mitochondrial energy metabolism. Electron microscopy directly confirmed these findings, and immunofluorescence revealed fragmented TOM20 signals, reflecting loss of mitochondrial network integrity. As mitochondria are the primary sites of fatty acid β-oxidation, their dysfunction results in increased electron leakage from the respiratory chain and a burst of ROS. Consistently, biochemical assays demonstrated markedly decreased SOD, CAT, and GSH-Px activities accompanied by elevated MDA levels at week 8, suggesting exhaustion of the hepatic antioxidant defense system. Of particular importance, MAMs provide a dynamic interface for ER–mitochondria crosstalk and metabolic coordination. In this study, electron microscopy revealed ER dilation and vesiculation occurring concurrently with mitochondrial injury. ER stress has been reported to induce calcium ion leakage, which may lead to mitochondrial calcium overload, thereby exacerbating ROS generation and potentially impairing mitochondrial function. This dysregulation of the mitochondria–ER axis may represent a mechanism underlying oxidative stress amplification [27,28,29]. The enrichment of the cysteine and methionine metabolism pathway in metabolomic analysis suggests that the organism may mobilize methionine and other amino acids to support GSH synthesis in order to counteract ROS; however, this compensatory defense may become insufficient at later stages, contributing to antioxidant exhaustion [30,31]. This phase is consistent with a pathological transition from simple steatosis to steatohepatitis, during which oxidative stress may shift from a compensatory response to a decompensated injury state.

Markers of oxidative stress and inflammation appeared closely coupled over the HFCD time course. From week 8 onward, hepatic TNF-α and IL-6 levels increased (2.8-fold and 3.1-fold, respectively), accompanied by stronger TNF-α immunofluorescence, supporting the emergence of an inflammatory response at the mid–late stage. These patterns are consistent with prior reports that excess ROS can engage NF-κB and/or NLRP3 inflammasome-related signaling to promote pro-inflammatory cytokine expression [32,33]. In addition, lipid accumulation may act as damage-associated molecular patterns (DAMPs) and contribute to activation of Kupffer cells and hepatic stellate cells (HSCs). Oxidative stress and inflammation have also been described as a feed-forward loop, in which TNF-α can impair mitochondrial respiratory chain function and thereby increase ROS generation, while ROS may further amplify inflammatory signaling [34,35]. Such interactions could become more prominent during weeks 8–10, coinciding with more severe hepatic injury. In the metabolomic analysis, enrichment of the KEGG term “central carbon metabolism in cancer” (which includes glycolysis- and TCA-related nodes) may reflect a shift in energy metabolism toward glycolytic processes, although this inference remains indirect without flux-level measurements. Such metabolic adjustments could plausibly contribute to redox imbalance and a pro-inflammatory microenvironment [36,37]. Overall, our data suggest that inflammatory activation is not only associated with metabolic dysregulation but may also contribute to the progression of fibrogenic remodeling.

Fibrosis is a key histopathological feature associated with progression of MASLD toward advanced liver disease. By week 10, Masson’s trichrome and Sirius Red staining showed increased collagen deposition in hepatic tissue, and α-SMA immunofluorescence was enhanced, consistent with activation of hepatic stellate cells (HSCs) and a myofibroblast-like phenotype accompanied by extracellular matrix accumulation. These changes likely reflect multifactorial metabolic–immune interactions rather than a single driver. For example, lipid peroxidation products (e.g., MDA) and pro-inflammatory/pro-fibrogenic mediators (e.g., TNF-α and TGF-β) have been reported to promote HSC activation. In addition, the metabolomic analysis identified an increase in LPC (19:0), which has been implicated as a pro-fibrotic lipid mediator and may influence HSC responses via GPCR-related signaling, potentially contributing to collagen production [38,39].

Ultrastructural alterations at the late stage were consistent with severe cellular injury. Electron microscopy showed mitochondrial vacuolization and membrane disruption, which may reflect profound mitochondrial damage and impaired energy handling; in parallel, ER structural alterations were observed, potentially indicating compromised proteostasis and disturbed calcium homeostasis. These morphological findings were broadly aligned with the metabolomics results, in which pathway enrichment suggested reduced representation of TCA-cycle-related and amino-acid–metabolism-related metabolites, supporting the possibility that hepatocytes had transitioned toward a more decompensated functional state. At this stage, fibrotic remodeling may extend beyond an adaptive reparative response and become associated with architectural distortion and progressive loss of hepatic function.

Time-resolved untargeted metabolomic analysis provided a systems-level view of metabolic remodeling during HFCD feeding. KEGG pathway enrichment suggested that differential metabolites were mainly annotated to pathways related to ABC transporters, amino acid biosynthesis/metabolism, nucleotide metabolism, and central carbon metabolism. Rather than indicating a single pathway defect, these enrichment patterns point to coordinated pathway-level associations. For example, enrichment of ABC transporters may reflect altered handling of xenobiotic-related metabolites, consistent with the observed changes in specific compounds (e.g., reduced paraoxon). Enrichment signals involving glycine/serine/threonine metabolism may be compatible with shifts in one-carbon-related metabolism, which could influence nucleotide homeostasis and redox buffering capacity; however, these links remain indirect without targeted validation. In addition, enrichment of nucleotide-metabolism terms may suggest altered demands for cellular turnover and repair in the injured liver microenvironment. Central carbon metabolism-related enrichment (including glycolysis/TCA-linked nodes) may reflect a shift in energy metabolism, although direct evidence for changes in flux between oxidative phosphorylation and glycolysis was not assessed in this study. With respect to potential upstream contributors, prior literature has linked choline availability to PC homeostasis (via the CDP-choline/Kennedy pathway and PEMT-dependent routes) and to methyl-group metabolism through SAM. In this context, HFCD feeding—incorporating choline restriction—may plausibly contribute to broad metabolic changes through combined effects on phospholipid handling and one-carbon metabolism [40,41]. Accordingly, our data support a working model in which the choline-deficient HFCD regimen can reproduce several metabolic features reported in human MASLD, while the precise causal contribution of choline deficiency versus high-fat feeding requires further substantiation [42].

Since Ludwig et al. [43] first described hepatic steatosis accompanied by inflammation and related histological alterations in 1980, NAFLD is widely acknowledged as a continuum of hepatic disorders, encompassing conditions from simple steatosis to nonalcoholic steatohepatitis (NASH), cirrhosis, and hepatocellular carcinoma. Traditionally, its diagnosis has relied on the presence of hepatic steatosis—defined as lipid accumulation in ≥5% of hepatocytes as assessed by biopsy or imaging—while excluding other etiologies such as excessive alcohol consumption and chronic liver diseases including viral hepatitis and drug-induced liver injury [2]. In 2020, to emphasize the central role of metabolic dysfunction, Eslam et al. proposed the term MAFLD, shifting the diagnostic paradigm from an exclusion-based to an inclusion-based framework. Under this definition, hepatic steatosis accompanied by overweight/obesity, diabetes, or metabolic abnormalities is sufficient for diagnosis, even in the presence of alcohol consumption [44]. Subsequently, the 2023 International Delphi Consensus further refined the nomenclature to MASLD, maintaining the metabolic foundation (hepatic steatosis plus ≥1 cardiometabolic risk factor) while redefining alcohol intake thresholds and redrawing disease boundaries [45]. This terminological evolution marks a conceptual shift from pathological description to a more explicitly metabolic-driven clinical framework, highlighting the need to elucidate the specific metabolic perturbations—such as altered choline availability and related pathways—that may contribute to hepatic fibrogenesis.

**Figure 8:**
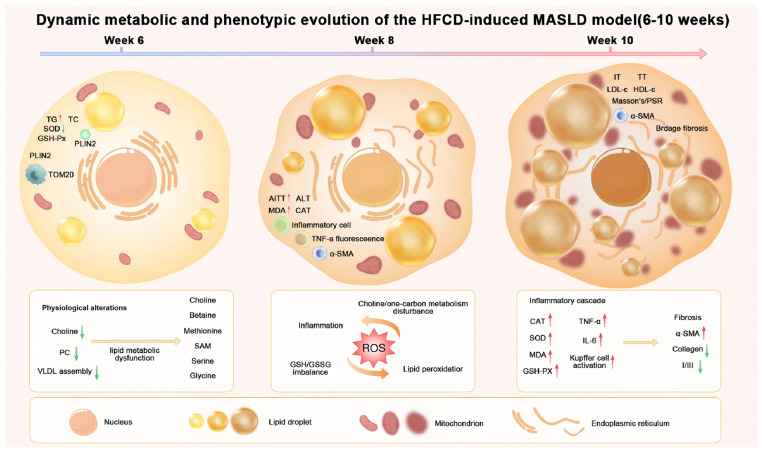
Spatiotemporal progression of HFCD-induced MASLD from weeks 6 to 10. Schematic overview of the stage-dependent pathological and metabolic changes during HFCD feeding. Week 6 is characterized by early steatosis with lipid droplet accumulation and mitochondrial perturbation. Week 8 shows aggravated oxidative stress and inflammatory activation with emerging stellate cell activation. By week 10, these alterations progress to pronounced fibrogenesis with collagen deposition and bridging fibrosis. Arrows indicate relative changes compared with controls.

Through a multimodal analytical platform, the present study systematically characterized the dynamic progression of MASLD induced by HFCD feeding. Our core findings suggest that HFCD feeding, which includes choline restriction, may serve as an early contributor to hepatic steatosis and is associated with an oxidative stress–inflammation–fibrosis cascade in the context of metabolic remodeling. Compared with the traditional MCD diet model—which induces rapid MASLD but is confounded by weight loss and has limited metabolic resemblance to humans—the HFCD model used in this study exhibited progressive weight gain, tendencies toward insulin resistance, and gradual fibrosis, features that are more broadly consistent with the natural course of human MASLD (Figure 8). Incorporating MRI–PDFF for noninvasive lipid quantification and dynamic immunofluorescence tracking of molecular markers such as α-SMA and TNF-α, this model may offer a practical and physiologically relevant platform for pharmacological evaluation and mechanistic studies. Moreover, we established a 6–10-week temporal progression map, which may provide a well-defined reference window for future interventional research. For instance, therapeutic interventions initiated at week 6 could be considered for evaluating anti-steatotic effects, whereas treatments introduced at week 8 might be better suited for assessing anti-inflammatory or anti-fibrotic efficacy. To further strengthen the generalizability and mechanistic resolution of the present time-resolved framework, several aspects merit follow-up. First, although the HFCD regimen is designed to restrict choline availability, serum choline and hepatic phosphatidylcholine (PC) were not directly quantified in this study; incorporating these measurements would better anchor the proposed choline-related axis. Second, only male mice were used, and whether females exhibit comparable phenotypes remains to be verified. Third, the untargeted metabolomics workflow provides a broad, semi-quantitative overview; targeted LC–MS/MS validation of representative metabolites (e.g., betaine and selected phospholipids) will strengthen the evidence chain and help prioritize key metabolite candidates and pathways for subsequent mechanistic testing.

## 5. Conclusions

HFCD feeding induces a temporally ordered transition from steatosis to fibrosis through choline-deficiency-associated metabolic impairment. Early oxidative stress and mitochondrial dysfunction precede inflammatory activation and stellate-cell engagement. Integrated biochemical, histological, imaging, and metabolomic analyses delineate a 6–10-week progression framework, identifying choline-related pathways as potential targets for early MASLD intervention.

## Figures and Tables

**Table 1:** Specific Composition of HFCD Feed.

Ingredients	Weight (kg)
Casein	2.6
Cystine	0.039
Maltodextrin	1.625
Sugarcane inch sugar	0.9464
Cellulose	0.65
Lard	3.185
Soybean oil	0.325
Mineral Mix S10026B	0.65
Choline tartrate	0
Vitamin Mix V10001C	0.013
Total weight	10.000

Carbohydrates account for about 20% of the energy supply, fat accounts for about 60%, and protein accounts for about 20%.

## Data Availability

The original contributions presented in this study are included in the article and Supplementary Materials. Further inquiries can be directed to the corresponding authors.

## Supplementary Materials

The following supporting information can be downloaded at: https://www.mdpi.com/article/10.3390/antiox15010110/s1. Supplementary Methods S1: Metabolite annotation workflow; Supplementary Methods S2: Database-assisted metabolite annotation workflow; Table S1: m/z–RT feature list and metabolite annotation details. Reference [46] is cited in the Supplementary Materials.

## Abbreviations

The following abbreviations are used in this manuscript:

ABC	ATP-binding cassette
ALT	Alanine aminotransferase
ANOVA	Analysis of variance
AST	Aspartate aminotransferase
BSA	Bovine serum albumin
CAT	Catalase
DAMPs	Damage-associated molecular patterns
DAPI	4′,6-diamidino-2-phenylindole
ELISA	Enzyme-linked immunosorbent assay
ER	Endoplasmic reticulum
FOV	Field of view
GSH-Px	Glutathione peroxidase
H&E	Hematoxylin and eosin
HDL-C	High-density lipoprotein cholesterol
HFCD	High-fat, choline-deficient diet
HMDB	Human Metabolome Database
HSCs	Hepatic stellate cells
IL-6	Interleukin-6
KEGG	Kyoto Encyclopedia of Genes and Genomes
LC-MS	Liquid chromatography–mass spectrometry
MAFLD	Metabolic dysfunction-associated fatty liver disease
MAMs	Mitochondria-associated membranes
MASLD	Metabolic dysfunction-associated steatotic liver disease
MASH	Metabolic dysfunction-associated steatohepatitis
MDA	Malondialdehyde
MRI	Magnetic resonance imaging
MRI-PDFF	Magnetic resonance imaging–proton density fat fraction
MS/MS	Tandem mass spectrometry
NAFLD	Nonalcoholic fatty liver disease
NASH	Nonalcoholic steatohepatitis
PC	Phosphatidylcholine
PCA	Principal component analysis
PDFF	Proton density fat fraction
PFA	Paraformaldehyde
PLIN2	Perilipin 2
PLS-DA	Partial least squares discriminant analysis
QC	Quality control
ROI	Region of interest
ROS	Reactive oxygen species
RT	Room temperature
SD	Standard deviation
SOD	Superoxide dismutase
SPF	Specific pathogen free
TCA	Tricarboxylic acid cycle
TC	Total cholesterol
TE	Echo time
TEM	Transmission electron microscopy
TG	Triglyceride
TNF-α	Tumor necrosis factor-alpha
TOM20	Translocase of outer mitochondrial membrane 20
TR	Repetition time
UPLC	Ultra-performance liquid chromatography
UV	Unit variance
VIP	Variable importance in projection
VLDL	Very low-density lipoprotein
XCMS	XCMS (LC–MS data processing software)
