# Supplementary material for: Choline Deficiency Drives the Inflammation–Fibrosis Cascade: A Spatiotemporal Atlas of Hepatic Injury from Weeks 6 to 10
Source: Antioxidants (Basel). 2026 Jan 15;15(1):110. doi: 10.3390/antiox15010110 (PMC12838149; doi:10.3390/antiox15010110)
Supplement: Supplementary file 1 [file antioxidants-15-00110-s001.zip › Supplementary Methods S1.pdf]

Supplementary Methods S1. Database-Assisted Metabolite Annotation and Identification Workflow

Step-by-step description prepared to address reviewer requests for transparency and reproducibility of metabolite assignments

1. Overview

This document summarizes the workflow used to preprocess LC–MS/MS data, annotate metabolites, and map the annotated metabolites to public databases (HMDB, METLIN, and KEGG). The aim is to provide a reader-friendly, reproducible guide that explains how the reported metabolite identities were obtained and how the reported m/z values and retention times (RT) relate to the final results.

2. Data acquisition and raw file conversion

LC–MS/MS platform. Polar metabolites were analyzed using a Vanquish UHPLC system coupled to an Orbitrap Exploris 120 high-resolution mass spectrometer. Data were acquired in both positive and negative ion modes with data-dependent MS/MS acquisition. These settings provided accurate MS1 masses together with MS/MS spectra for downstream annotation.

Raw data conversion. Raw vendor files were converted to mzXML format using ProteoWizard (v3.0.24054). The mzXML files were used as input for subsequent feature extraction and alignment.

3. Feature extraction, quality control, and preprocessing

Feature detection, alignment, and integration were performed using an in-house R pipeline based on XCMS. A pooled quality-control (QC) sample was prepared by mixing equal aliquots of all study samples and was injected across the analytical sequence to monitor signal stability.

- Feature extraction and alignment were conducted across all samples to generate an initial feature table (m/z–RT features).
- QC-based filtering was applied to remove unstable signals (based on relative standard deviation, RSD/CV, in QC injections).
- Features with excessive missingness were removed (e.g., features with >50% missing values within a group or across all groups).

- Remaining missing values were imputed using one-half of the minimum positive value for each feature.
- Signal normalization was performed using internal standards included in the extraction solvent; global intensity normalization (e.g., TIC-based scaling) was used as implemented in the analysis pipeline to mitigate systematic drift.
- A log transformation and scaling were applied prior to multivariate modeling (PCA/OPLS-DA) to reduce heteroscedasticity and the influence of high-variance variables.

4. Database-assisted metabolite annotation

Metabolite annotation was performed for features with acquired MS/MS spectra. For each feature, the measured precursor m/z, retention time (RT), ionization mode, and MS/MS spectrum were recorded. Candidate identities were then generated and ranked using accurate mass matching and MS/MS spectral similarity against public resources (HMDB and METLIN), followed by identifier mapping to KEGG for pathway analysis.

4.1. Candidate generation using accurate mass (MS1)

Accurate mass matching was used to generate candidate metabolites. The precursor m/z was queried against HMDB and METLIN within a predefined mass tolerance consistent with the reported MS1 mass error (ppm). Ion mode and common adduct forms were considered during candidate generation (e.g., $[M+H]^+$, $[M+Na]^+$ in positive mode; $[M-H]^-$ in negative mode).

4.2. MS/MS spectral matching and scoring

For each candidate, experimental MS/MS spectra were compared with reference spectra in HMDB/METLIN (and, where applicable, a local reference library). A spectral similarity score (reported as MS2 score) was used to rank candidates; higher scores indicate better agreement between experimental and reference fragment patterns. Candidate ranking considered (i) MS/MS similarity, (ii) MS1 mass error (ppm), and (iii) RT agreement when RT information was available in a reference library.

4.3. Retention time (RT) as orthogonal evidence

Retention time was used as an orthogonal evidence layer to support metabolite identities. When authentic standards or curated RT entries were available, RT matching was used to increase confidence. When standards were not available, RT is still reported for transparency and to facilitate independent verification; in such cases, isomeric ambiguity cannot be fully excluded.

4.4. Mapping to KEGG for pathway analysis

After an identity was assigned, database identifiers (HMDB ID and KEGG compound ID) were recorded whenever available. For pathway enrichment, annotated metabolites were mapped to Mus musculus KEGG pathways using KEGG compound identifiers. If multiple database

candidates existed for a single feature, assignments were treated as putative and were handled cautiously in downstream biological interpretation.

4.5. Annotation confidence levels (MSI)

Annotation confidence was described using the Metabolomics Standards Initiative (MSI) framework (Sumner et al., 2007).

MSI Level	Evidence basis (summary)
Level 1	Confirmed identification: matched to an authentic reference standard by accurate mass, MS/MS spectrum, and RT.
Level 2	Putatively annotated compound: supported by accurate mass and MS/MS spectral similarity to public or curated libraries.
Level 3	Putatively characterized compound class: confident compound class assignment, but exact structure not confirmed.
Level 4	Unknown: detectable feature without reliable structural assignment.

In this study, most reported metabolites were annotated at MSI Level 2, and any higher-confidence Level 1 identifications (if applicable) require RT agreement with authentic standards or a curated RT library.

5. Step-by-step guide to reproduce the reported metabolite assignments

The practical steps below describe how a reader can trace each reported metabolite assignment from the feature table to database evidence:

1. Locate the metabolite/feature of interest in Supplementary Table S1 using its unique ID or metabolite name.
2.Record the measured precursor m/z, RT, ion mode, and MS1 mass error (ppm) provided in the table.
3. Query HMDB and/or METLIN using the precursor m/z within the reported ppm tolerance and the appropriate ion mode/adduct assumptions.
4. For the shortlisted candidates, compare the experimental MS/MS spectrum (as represented by the MS2 match and score) with reference spectra; prioritize candidates with the highest MS2 score and chemically plausible fragments.

5. Where RT reference information is available, verify that the measured RT is consistent with the expected RT under the reported LC conditions. If RT reference is unavailable, interpret the assignment as putative and note potential isomeric ambiguity.
6. Retrieve/confirm HMDB ID and KEGG compound ID for the selected candidate; use KEGG IDs for pathway mapping and enrichment analysis.
7. For transparency and re-analysis, retain both the full feature list and the subset of reported metabolites together with the key metadata (m/z, RT, ppm error, MS2 score, and annotation level).

6. Reporting in Supplementary Table S1

Supplementary Table S1 provides the measured m/z values and retention times (RT) for the reported metabolites and includes additional metadata that supports assessment of identification confidence (e.g., MS2 score and annotation level). Columns include metabolite name (MS2 name), MS2 score, annotation level, MS1 mass error (ppm), measured m/z, RT, ion mode, and (where applicable) statistical outputs used in downstream analyses (e.g., VIP, P-value, Q-value).

7. Notes on interpretation and limitations

Untargeted metabolomics provides putative annotations for many compounds. Even with accurate mass and MS/MS matching, some features may correspond to structural isomers that share similar fragments. Where authentic standards are unavailable, identifications should be interpreted as putative (MSI Level 2) and used primarily for pathway-level interpretation.

References

Sumner LW, Amberg A, Barrett D, et al. Proposed minimum reporting standards for chemical analysis. Metabolomics Standards Initiative (MSI). Metabolomics. 2007;3(3):211–221.
