# Supplementary material for: Choline Deficiency Drives the Inflammation–Fibrosis Cascade: A Spatiotemporal Atlas of Hepatic Injury from Weeks 6 to 10
Source: Antioxidants (Basel). 2026 Jan 15;15(1):110. doi: 10.3390/antiox15010110 (PMC12838149; doi:10.3390/antiox15010110)
Supplement: Supplementary file 1 [file antioxidants-15-00110-s001.zip › Supplementary Methods S2.pdf]

Metabolite Extraction:

1. Metabolite Extraction from Animal Tissues:

Animal tissue samples (25 mg ± 1 mg) were mixed with beads and 500 µL of extraction solvent (MeOH:ACN:H2O = 2:2:1, v/v/v) containing deuterated internal standards and vortexed for 30 s.

The mixtures were homogenized (35 Hz, 4 min) and sonicated for 5 min in a 4 °C water bath; this step was repeated three times.

Samples were incubated at -40 °C for 1 h to precipitate proteins and then centrifuged at 12,000 rpm (RCF = 13,800 × g; R = 8.6 cm) for 15 min at 4 °C. The supernatant was transferred to fresh glass vials for analysis. A pooled quality control (QC) sample was prepared by mixing equal aliquots of the supernatants from all samples.

2. LC-MS/MS Analysis:

For polar metabolites, LC-MS/MS analyses were performed using an UHPLC system (Vanquish, Thermo Fisher Scientific) equipped with a Waters ACQUITY UPLC BEH Amide column (2.1 mm × 50 mm, 1.7 µm) coupled to an Orbitrap Exploris 120 mass spectrometer (Thermo Fisher Scientific). The mobile phase consisted of 25 mmol/L ammonium acetate and 25 mmol/L ammonia hydroxide in water (pH = 9.75) (A) and acetonitrile (B). The autosampler temperature was 4 °C, and the injection volume was 2 µL. The Orbitrap Exploris 120 mass spectrometer was operated in information-dependent acquisition (IDA) mode under the control of Xcalibur software (Thermo Fisher Scientific). In this mode, the acquisition software continuously evaluates the full-scan MS spectrum. The ESI source conditions were set as follows: sheath gas flow rate, 50 Arb; auxiliary gas flow rate, 15 Arb; capillary temperature, 320 °C; full MS resolution, 60,000; MS/MS resolution, 15,000; collision energy, SNCE 20/30/40; spray voltage, 3.8 kV (positive) or -3.4 kV (negative), respectively.

2.1. Data preprocessing and annotation:

Raw data were converted to mzXML format using ProteoWizard and processed with an in-house R program based on XCMS for feature detection, extraction, alignment, and integration. Relevant R packages were applied for metabolite identification.

Reference: Zhou, Z., Luo, M., Zhang, H., Yin, Y., Cai, Y., & Zhu, Z. J. (2022). Metabolite annotation from knowns to unknowns through knowledge-guided multi-layer metabolic networking. Nature Communications, 13(1), 6656.

2.2. Data analysis:

In this study, 47,051 features were detected and 2,652 metabolites remained after relative standard deviation (RSD) de-noising. Missing values were imputed with half of the minimum value. Internal standard normalization was applied. The final dataset containing the feature number, sample name, and normalized feature area was imported into SIMCA 18.0.1 (Sartorius Stedim Data Analytics AB, Umea, Sweden) for multivariate analysis. Data were scaled and log-transformed to minimize the impact of both noise and high variance. Principal component analysis (PCA), an unsupervised dimensionality-reduction method, was performed to visualize sample distribution and grouping. The 95% confidence interval in the PCA score plot was used to identify potential outliers in the dataset.

To visualize group separation and identify significantly changed metabolites, supervised orthogonal projections to latent structures discriminant analysis (OPLS-DA) was applied. A 7-fold cross-validation was performed to calculate R2 and Q2. R2 indicates how well the variation of a variable is explained and Q2 indicates predictive ability. To assess the robustness and predictive ability of the OPLS-DA model, 200 permutation tests were further conducted. The R2 and Q2 intercept values were obtained. Here, a smaller Q2 intercept value indicates a lower risk of overfitting and a more robust model.

Furthermore, variable importance in the projection (VIP) values of the first principal component in the OPLS-DA model were calculated to summarize each variable's contribution. Metabolites with VIP > 1 and $p < 0.05$ (Student's t-test) were considered significantly changed metabolites.

In addition, commercial databases including KEGG (http://www.genome.jp/kegg/) and MetaboAnalyst (http://www.metaboanalyst.ca/) were used for pathway enrichment analysis.

3. Instruments used in this study

Instrument	Model	Manufacturer / Brand
Ultra-high performance liquid chromatography (UHPLC) system	Vanquish	Thermo Fisher Scientific
High-resolution mass spectrometer	Orbitrap Exploris 120	Thermo Fisher Scientific
Centrifuge	Heraeus Fresco 17	Thermo Fisher Scientific
Analytical balance	BSA124S-CW	Sartorius
Ultrasonic cleaner (ultrasonic bath)	PS-60AL	Shenzhen Leidbang Electronics Co., Ltd.
Homogenizer (tissue homogenizer)	JXFSTPRP-24	Shanghai Jingxin Technology Co., Ltd.
Freeze dryer (lyophilizer)	LGJ-10C	Sihuan Foring Instrument Technology Development Co., Ltd.
